# Effects of Oral Administration of Silymarin in a Juvenile Murine Model of Non-alcoholic Steatohepatitis

**DOI:** 10.3390/nu9091006

**Published:** 2017-09-12

**Authors:** Veronica Marin, Silvia Gazzin, Sabrina E. Gambaro, Matteo Dal Ben, Sonia Calligaris, Monica Anese, Alan Raseni, Claudio Avellini, Pablo J. Giraudi, Claudio Tiribelli, Natalia Rosso

**Affiliations:** 1 Fondazione Italiana Fegato ONLUS-Centro Studi Fegato, Area Science Park Basovizza Bldg, Q SS 14 Km 163,5, Basovizza, 34149 Trieste, Italy; veronica.marin@fegato.it (V.M.); silvia.gazzin@fegato.it (S.G.); sabrigambaro@gmail.com (S.E.G.); mdalben@fegato.it (M.D.B.); pablo.giraudi@fegato.it (P.J.G.); ctliver@fegato.it (C.T.); 2 Università di Udine, Dipartimento di Scienze AgroAlimentari, Ambientali e Animali, Via Sondrio 2/A, 33100 Udine, Italy; sonia.calligaris@uniud.it (S.C.); monica.anese@uniud.it (M.A.); 3 IRCCS Burlo Garofolo Paediatric Hospital, Clinical Chemistry Laboratory, 34100 Trieste, Italy; alan_ciop@yahoo.it; 4 Azienda Ospedaliero-Universitaria “Santa Maria della Misericordia”, Dipartimento di Laboratorio, Istituto di Anatomia Patologica, 33100 Udine, Italy; claudio.avellini@asuiud.sanita.fvg.it

**Keywords:** NAFLD, NASH, fibrosis, silymarin, in vivo model, in vitro model, therapeutic approach

## Abstract

The increasing prevalence of non-alcoholic fatty liver disease (NAFLD) in adolescents is challenging the global care system. No therapeutic strategies have been defined so far, and changes in the lifestyle remain the only alternative. In this study, we assessed the protective effects of silymarin in a juvenile non-alcoholic steatohepatitis (NASH) model and the in vitro effects on fat-laden human hepatocytes. C57Bl/6 mice were exposed to HFHC diet immediately after weaning. After eight weeks, animals showed histological signs of NASH. Silymarin was added to the HFHC diet, the treatment continued for additional 12 weeks and the effects on BMI, hepatomegaly, visceral fat, lipid profile, transaminases, HOMA-IR, steatosis, inflammation, fibrosis, oxidative stress, and apoptosis were determined. The switch from HFHC to control diet was used to mimic lifestyle changes. In vitro experiments were performed in parallel in human hepatocytes. HFHC diet supplemented with silymarin showed a significant improvement in glycemia, visceral fat, lipid profile, and liver fibrosis. Moreover, it reduced (both in vitro and in vivo) ALT, hepatic inflammation, oxidative stress, and apoptosis. Lifestyle changes restored the control group parameters. The data presented show the beneficial effects of the oral administration of silymarin in the absence of changes in the dietary habits in a juvenile model of NASH.

## 1. Introduction

The increasing prevalence of non-alcoholic fatty liver disease (NAFLD) worldwide is becoming a challenge for the modern global care system [1]. While NAFL is a reversible and benign disorder, the progression towards the more severe non-alcoholic steatohepatitis (NASH) must be not disregarded. The booming prevalence of obesity and type 2 diabetes mellitus in young age and adolescence [2,3] contributes not only to the onset of the condition but also provides more time for its evolution. Therefore NASH incidence in children and adolescents represents the major threat to the upcoming years. To date, there is no consensus concerning an effective pharmacological treatment for NASH, and the only recomended treatment relies on lifestyle modifications [4] (diet [5] and physical activity [6,7,8]). However, the lack of compliance is still the main obstacle to overcome. Currently, drug interventions are based on the association of several compounds as an attempt to reverse the co-morbidities of the metabolic syndrome. The molecular mechanisms leading to fat accumulation, oxidative imbalance, and liver fibrosis are the targets of the main classes of drugs usually associated with lifestyle modifications. Unfortunately, to date none of the proposed drugs have provided solid results. Thus, there is an evident need for the characterization of new therapeutic alternatives. Recent studies of food bioactive compounds represent an attractive approach to be considered. Silymarin, extracted from plant seeds and fruits of silybum marianum (commonly known as milk thistle [9,10]), has been used as medicinal herb from as early as fourth century B.C. It has been widely employed in the treatment of various liver disorders [11,12,13] due to its hepatoprotective properties such as anti-inflammatory [14], anti-proliferative [15,16,17], immunomodulatory [18], and anticholesterolemic properties [19,20]. In particular, silymarin has been widely proposed in the treatment of NASH although definitive data have not been provided so far. Due to its low bioavailability [21,22], some solubilizing compounds (phosphatidycholine; β-cyclodextrin, vitamin E) were added to the plant extracts to enhance intestinal absorption [23]. Although data obtained in animal models [24] are promising, the administration and the dosage are often difficult to be reproduced in humans. On the other hand, data from clinical trials using silymarin [25,26] are controversial and the real efficacy has been questioned for years. The main limitations are the lack of silymarin standardization among its various formulations and the still undefined effective dosage [27]. This situation is even worse among the pediatric and juvenile population, where the available information is scarce. Studies of dietary supplements in children have shown inconsistent effects to benefit children with NAFLD [28].

A good therapeutic approach to overcome the reticence towards lifestyle changes would be the use of silymarin as a dietary supplement. Thus, the aim of the present study is to assess the hepatoprotective effects of silymarin in an in vivo mouse model of juvenile NASH [29] and to determine the direct effect on human fat-laden hepatocytes in an in vitro model [30]. The novelty of our work relies on the use of a juvenile model of NASH (where males and females were considered as different experimental groups) to assess the effect of silymarin administrated orally, incorporated in the western diet, and in vitro together with free-fatty acids (FFA) as a strategy to prevent/revert the damage related to the high fat and high carbohydrate (HFHC) diet.

## 2. Materials and Methods

### 2.1. Experimental Set-Up

#### 2.1.1. Animal Model (In Vivo)

**Scheme 1:**
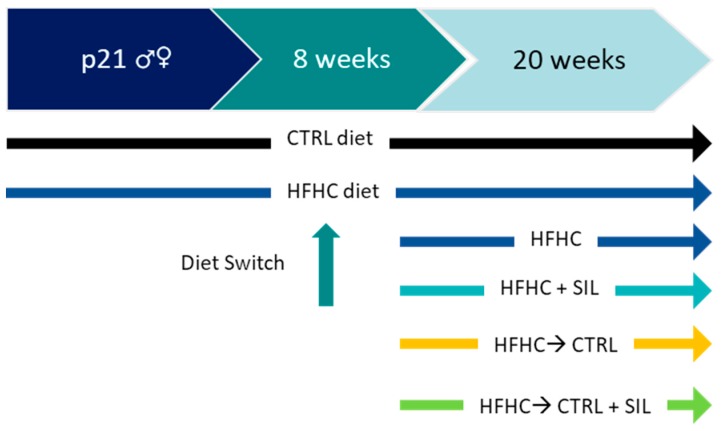
In vivo experimental set-up. The experimentation started at p21 (after weaning), animals were divided in control (CTRL) (black arrow) and high-fat high-carbohydrates (HFHC) (blue arrows) diet. After eight weeks, a group of animals continued with HFHC, whereas in others HFHC diet was switched to silymarin-formulated HFHC (HFHC + SIL) (cyan arrow), to CTRL (HFHC→CTRL) diet (orange arrow) and to CTRL plus the addition of silymarin (HFHC→CTRL + SIL) (green arrow). The trial continued until week 20.

C57Bl/6 mice pups (28 of each sex) were obtained from Harlan Laboratories S.R.L. (S.Piero al Natisone, Italy). Immediately after weaning, animals were randomly group-housed in cages based on the final experimental protocol (Scheme 1) in a temperature-controlled environment (22 °C ± 2 °C) and on a 12 h light/dark schedule, and fed ad-libitum with control diet (CTRL, D12328, Research Diets, New Brunswick, NJ, USA) or HFHC diet (HFHC: D12331, Research Diets, New Brunswick, NJ, USA) plus 42 g/L fructose/sucrose in drinking water, for a total of eight weeks (time required for the onset of NASH, as described previously [29]). After eight weeks, a group of animals continued with the HFHC; another group was fed with HFHC silymarin-enriched diet (added into the food pellet (33 mg/kg/diet) (HFHC + SIL) (see section below)), in a third group diet was switched from HFHC to CTRL diet (HFHC→CTRL) which represent the alimentary lifestyle changes, and in a fourth group the switch of diet from HFHC to CTRL was enriched with silimarin (HFHC→CTRL + SIL) to assess and eventual synergic effect of silymarin with lifestyle changes . The experimentation then continued for a further 12 weeks (Scheme 1). Animals were sacrificed at the end of the trial. After O/N fasting, animals were deeply anesthetized (Zoletil 10 mg/kg and Xilazine 5 mg/kg of body weight, intra-peritoneal). Blood was collected by cardiac puncture (exsanguination). Whole blood was allowed to clot at room temperature (RT) for 20 min, followed by centrifugation at 3500× *g* for 5 min at RT to separate the serum. All experimental protocols were approved by the local committee of the Medical Research Institute and by the National Authority (Ministero della Salute-Direzione Generale della Sanità Animale e dei Farmaci Veterinary—Ufficio VI, Rome, Italy. Approval 11072013). They are in accordance with FELASA guidelines and the National law for Laboratory Animal Experimentation (D. lgs 116/92, article 7). The maximal effort was made to reduce the number of animals used and their sufferance in the respect of the 3R rule (Reduction, Refinement and Replacement).

#### 2.1.2. HFHC + SILymarin Food Formulation

To follow a physiological administration, silymarin extract (Sigma Aldrich, St. Luois, MO, USA) containing 210 mg/g of silybin was added into the HFHC diet and administrated orally. Briefly, the composition of the animal food provided by Research Diets Inc. (Research Diets, New Brunswick, NJ, USA) and designed for a high fat high carbohydrates diet (HFHCD) was modified opportunely to include silymarin in the product (D12331, 23% protein, 35.5% carbohydrate, 35.8% fat in form of soybean oil and hydrogenated coconut oil). In particular, silymarin powder (0.01 g/g) and Tween 80 (Sigma Aldrich, St. Luois, MO, USA) (0.1 g/g) were added to coconut oil (Unigrà, Conselice, Italy) previously heated at 40 °C. The system was maintained at 40 °C until silymarin complete solubilization. The oil enriched with silymarin was manually incorporated at a concentration of 14% by weight in paste obtained by grounding the animal feed pellet with a mixer (Kenwood, Prospero KM260, Havant, UK). Subsequently, silymarin-enriched paste was subdivided in aliquots of 4.5 g cylinders. The effective concentration of the bioactive principle (silybin) contained in each food cylinder was (270 mg/kg) determined by HPLC analysis.

#### 2.1.3. Cellular In Vitro Model

Silibinin is the major component (50–60%) of the silymarin extract and is characterized by an equimolar combination of two diasteroisomers, called silybin A and B [31]. The use of mice primary hepatocytes was excluded as an attempt to reduce the number of animals in the experimentation. Thus, the translation of the direct effect of silybin on human hepatocytes was assessed in vitro, by using stabilized hepatic cell lines. The hepatic cell line (HuH7) (JHSRRB, Cat#JCRB0403), obtained from the Health Science Research Resources Bank (Osaka, Japan), was maintained in culture using Dulbecco’s Modified Eagle’s high glucose Medium (DMEM), supplemented with 10% *v/v* fetal bovine serum, 2 mM l-glutamine, 10,000 U/mL penicillin and 10 mg/mL streptomycine, at 37 °C, under 5% CO_2_ and in a 95% humidified atmosphere. Mycoplasma contamination has been excluded by testing the cultures periodically using fluorescent staining (Hoechst 33258). For the induction of NAFLD, cells were exposed for 24 h to 1200 µM of a mixture of FFA (palmitic:oleic in 1:2 molar ratio, respectively) as previously described [30]. Silybin (Cat#1040 Extrasynthese, Genay, France) 10 mM stock solution was prepared in DMSO. In order to assess silybin properties, cells were co-treated with FFA (1200 µM) and different concentrations (1, 5, 7.5 µM) of silybin for 24 h. The minimal silybin concentration able to reduce the parameters under study was 5 µM, thus this was the experimental concentration used in all the cell treatments. The obtained data was compared vs. FFA-treated cells. For the preparation of both FFA and FFA + silybin (FFA + SIL) DMSO was used as vehicle, so cells exposed to vehicle are referred as Control (CTRL) in all graphs.

### 2.2. Intracellular Fat Quantification

Intracellular fat content in vitro was determined fluorometrically based on Nile Red staining, a vital lipophilic dye used to label fat accumulation in the cytosol [32,33]. After 24 h of FFA exposure, adherent monolayer cells were washed twice with PBS and detached by tripsinization. After a 5 min centrifugation at 1500 rpm, the cell pellet was resuspended in 3 mL of PBS and incubated with 0.75 μg/mL Nile Red dye for 15 min at room temperature. Nile Red intracellular fluorescence was determined by flow cytofluorometry using a Becton Dickinson FACS Calibur System on the FL2 emission channel through a 585 ± 21 nm band pass filter, following excitation with an argon ion laser source at 488 nm. Data were collected in 10,000 cells and analyzed using Cellquest software from BD Biosciences (San Jose, CA, USA).

### 2.3. Body, Liver, Perigonadal Adipose Tissue Weight, and Food Intake

Body weight was recorded weekly, while liver and perigonadal (epidydimal/ovarian) fat pad tissues were dissected and weighed only at the end of the trial. Moreover, sacrificed animals’ body weight and naso-anal length were recorded for indirect computation of body composition via body mass index (BMI), for the confirmation of the development of the obese phenotype [34]. To exclude an eventual effect of food-intake after the diet switch, the grams of consumed food (and the equivalent Kcal) were recorded.

### 2.4. Assessment of Glucose Homeostasis, Serum Insulin, and Insulin Resistance

Glycemia and insulinemia were measured after 6 h fasting every four weeks (two days before sacrifice), collecting few blood drops from submandibular vein (about 40 μL per animal) in local lidocaine anesthesia. Glucose was measured in whole blood using One Touch verio IQ^®^ meter (Life Scan Europe, Zug, Switzerland) accordingly to manufacturer’s instructions. Serum insulin content was quantified from the same blood sample by AlphaLISA Insulin Kit (Perkin Elmer, Waltham, MA, USA) following manufacturer’s instructions. The homeostasis model assessment of insulin resistance HOMA-IR was calculated for each animal according to the following formula: blood glucose (mg/dL) × fasting insulin (μU/mL)/405 [35].

### 2.5. Biochemical Parameters

Total cholesterol (Chol), high-density lipoprotein cholesterol (HDL-C), and low density lipoprotein cholesterol (LDL-C) contents were assessed. As a measurement of liver damage, serum alanine (ALT) activity was also quantified. Enzymatic colorimetric kits (Roche Diagnostics GmbH; Mannheim, Germany) were used to measure these parameters in a Roche HITACHI Cobas e501 instrument (Roche Diagnostics GmbH; Mannheim, Germany) accordingly to manufacturer’s instructions by enzymatic colorimetric assay (Roche Diagnostics GmbH; Mannheim, Germany). ALT/AST activity in cell supernatants upon the treatments described before, were assessed by Alanine Aminotransferase Activity Assay Kit and Aspartate Aminotransferase Activity Assay Kit (Sigma-Aldrich^®^, St. Louis, MO, USA). The amount of pyruvate and glutamate quantified with these colorimetric kits are respectively proportional to ALT and AST enzymatic activity in each sample. The assay and the kinetic measurements were performed following manufacturer’s instructions. The absorbance for ALT was measured at 570 nm and for AST at 450 nm using an EnSpire^®^ Multimode Plate Reader (Perkin Elmer).

### 2.6. Histopathological Analysis

At the end of the trial, the liver was harvested and a portion of tissue immediately fixed in buffered formalin (formaldehyde 4%, NaH_2_PO_4_ 4 g/L, Na_2_HPO_4_ 6.5 g/L: pH 6.8). Tissue sections were cut at a thickness of 3.5 μm and stained with Hematoxylin & Eosin (H&E) and Gömöri trichrome stain. Histology was read by a single independent pathologist, blinded to experimental design, and treatment groups. Scores were attributed to steatosis (0–3), lobular inflammation (0–3), portal inflammation (0–1), and ballooning (0–2). Fibrosis was analyzed separately with a scale from 0 to 4 according to Kleiner–Brunt Classification [36,37].

### 2.7. Collagen Determination after SIRIUS Red/Fast Green Staining

The collagen content of the liver was assessed by Lopez-De Leon and Rojkind [38] colorimetric method, using Sirius Red for the staining of collagenous proteins and Fast Green for non-collagenous proteins. The liver was harvested and a portion of tissue immediately fixed in buffered formalin (formaldehyde 4%, NaH_2_PO_4_ 4 g/L, Na_2_HPO_4_ 6.5 g/L: pH 6.8). Tissue sections were cut at a thickness of 3.5 μm and layered on glass slides. Slices were deparaffinized after incubation with xylenes (2 × 5 min), ethanol 100% (2 × 2 min), ethanol 95% (2 × 2 min), ethanol 70% (1 × 2 min), ethanol 50% (1 × 2 min), and distilled water (2 × 2 min). The slices were stained with Picro-Sirius Red Stain solution (0.1% direct red 80, 0.1% fast green FCF dissolve in 1.2% aqueous picric acid solution) and incubated for 60 min. After the staining, the slides were rapidly dipped in acidified water to destain and finally quickly dehydrate and mount in xylene. Absorbance of stained Sirius Red collagen was quantified after dye elution with 1 mL of 0.1% NaOH in absolute methanol (1:1). The eluted color was read immediately in a Beckman DU 640B spectrophotometer at 540 and 605 nm, the maximal absorbance of Sirius Red and Fast Green FCF respectively. In order to calculate the concentration of collagen we followed the procedure reported by Lopez-De Leon and Rojkind.

### 2.8. Oxidative Stress Determination

#### 2.8.1. Assessment of Lipid Peroxidation (MDA)

Malondialdehyde (MDA) is one of many low molecular weight end-products of lipid hydoperoxide decomposition and is the most often measured as an index of lipid peroxidation [39]. Thus, oxidative stress was assessed in vivo by measuring MDA, through the generation of colored products, after the reaction with thiobarbituric acid (TBA). Hepatic tissue was cut by medical instruments and homogenized on ice, using 10 mM Sodium Phosphate Na_3_P0_4_, 1 mM EDTA, 0.2 mM BHT buffer. The homogenate was centrifuged at 10,000× *g*, 4 °C for 10 min and supernatant was collected for MDA detection. Briefly, each sample was prepared by adding TBA (0.7% *w/v*) BHT (2% *w/v* in ethanol) SDS 8.1% and acetic acid 10% *v/v*. After 45 min heating at 100 °C pink pigments were formed. After a quick cooling process, samples were centrifuged at 10,000× *g* for 5 min. The supernatant was collected and added to a same volume of TCA 10%, in order to precipitate the proteins. The solution was vortexed and centrifuged at 15,000× *g* for 3 min. The supernatant was collected for absorbance detection at 440, 532, and 600 nm. MDA was calculated using Hodges et al. equation [40].

#### 2.8.2. GSH/GSSG

Reduced glutathione (GSH) is considered one of the most important scavengers of reactive oxygen species (ROS), and its ratio with oxidized glutathione (GSSG) may be used as a marker of oxidative stress [41]. Total glutathione (GSH) was determined in vivo by an enzymatic recycling procedure: the sulphydryl group of the molecule reacts with 5,5′-dithiobis-2-nitrobenzoic acid (DTNB, Ellman’s reagent) (Sigma-Aldrich, St. Louis, MO, USA) producing a yellow colored 5-thio-2-nitrobenzoic acid (TNB), and the disulfide is reduced by NADPH in the presence of glutathione reductase.

Hepatic tissue was homogenized on ice in percloric acid solution 5%. The homogenate was centrifuged at 10,000× *g*, 4 °C for 10 min and the acid-soluble fraction was collected and neutralized with 2 M KHCO_3_. After a centrifugation to remove the formed potassium perchlorate, the supernatant was divided to quantify both GSH and GSSG. For GSH detection, the sample was mixed with 6 mM DTNB and 0.3 mM NADPH. After incubation at 37 °C for 3 min, 200 U/mL glutathione reductase was added and immediately the rate of produced TNB was read at 415 nm using the EnSpire^®^ Multimode Plate Reader (Perkin Elmer, Waltham, MA, USA). Glutathione concentrations were calculated using appropriate standards and normalized by µg of proteins.

#### 2.8.3. Quantification of Reactive Oxygen Species (ROS)

ROS was measured in vitro by the use a cell permeable fluorogenic compound, H_2_DCFDA, which is easily taken up by cells. After intracellular cleavage of the acetyl groups, this molecule becomes substrate of the intracellular reactive species and may be oxidized to 2′,7′-dichlorofluorescein (DCF), fluorescent product. 2 × 10^4^ cells/cm^2^ were seeded in a 6-well plate and treated as described before. After FFA and FFA+SIL exposure for 1 h and 24 h, adherent cells were washed with PBS, and were loaded with 10 µM H_2_DCFDA, freshly prepared solution, for 1 h at 37 °C. Afterwards, cells were lysed with 500 µL of non-ionic detergent Triton X-100 0.2% in PBS. The fluorescence of all samples was quantified, using the EnSpire^®^ Multimode Plate Reader (Perkin Elmer); the excitation wavelength (λ_ex_) of DCF is 505 nm, and the emission wavelength (λ_em_) is 525 nm. Fluorescence was normalized by µg of protein assessed using fluorescamine, a non-fluorescent compound that rapidly reacts with primary amines in proteins to generate highly fluorescent pyrrolinone type moieties [42]. Using a standard curve, based on BSA serial dilutions, was calculated the amount of total protein (µg) for each sample. Data was expressed as AUF/µg proteins.

### 2.9. RNA Extraction and cDNA Synthesis

For obtaining total RNA, cells and tissues were lysed with of EuroGOLD RNAPure™ (EuroClone^®^) following the manufacturer’s instructions. Quantification was performed spectrophotometrically at 260 nm in a Beckman DU 640B spectrophotometer, using quartz cuvettes. The RNA purity was evaluated by measuring the ratio A260/A280, considering RNA with appropriate purity those showing values between 1.8 and 2.0; its integrity was evaluated by gel electrophoresis. The integrity of RNA was assessed on standard 1% agarose/formaldehyde gel. Isolated RNA was resuspended in RNAse free water and stored at −80 °C until analysis. Total RNA (1 μg) was reverse transcribed using High Capacity cDNA Reverse Transcription Kit (Applied Biosystems) according to manufacturer’s instructions. Retrotranscription was performed in a Thermal Cycler, following temperature protocol proposed by manufactures: 10 min at 25 °C (annealing), 120 min at 37 °C (cDNA synthesis), 5 min at 85 °C (enzyme denaturation).

### 2.10. Gene Expression Analysis by Real Time RT-PCR

cDNA samples obtained from RT-PCR were used in the Real Time quantitative PCR to determine gene expression after treatment. PCR amplification was carried out in 15 µL reaction volume containing cDNA, 1× iQ SYBR Green Supermix, and specific sense and anti-sense primers. The exception was TNF-α, whose amplification was carried out in 50 µL reaction volume.

All primer pairs used for Real Time PCR were designed using the software Beacon Designer 8.10 and they were synthesized by Sigma Genosys Ltd. Primer sequences, concentrations, and ng of cDNA used are reported in Table 1. Standard curves were prepared using a ‘calibrator’ cDNA for each target and reference gene. In order to verify the specificity of the amplification, a melt-curve analysis was performed, immediately after the amplification protocol. Non-specific products of PCR were found in any case. The relative quantification was made using the Pfaffl modification of the ΔΔCt equation [43], taking into account the efficiencies of individual genes. The results were normalized in vivo to *β-actin* and *Gapdh*, and in vitro to *β-actin* and ribosomal subunit *18S*, used as reference genes. The data were analyzed using iQ5™ optical system software Version 2.0 (Bio-Rad Laboratories Hercules, CA, USA).

### 2.11. Assessment of Apoptosis

#### 2.11.1. In Vivo

Evidence of chromatin condensation, a marker of cell death by apoptosis, was obtained by staining with Hoechst 33258 (Sigma Aldrich, St. Louis, MO, USA) on formalin embeded liver section. In brief, slices were deparaffinized after incubation with xylenes (2 × 5 min), ethanol 100% (2 × 2 min), ethanol 95% (2 × 2 min), ethanol 70% (1 × 2 min), ethanol 50% (1 × 2 min), and distilled water (2 × 2 min). The slices were then stained with 1 μg/mL Hoechst 33258 and incubated for 30 min. After the staining, the slides were rapidly dipped in acidified water to destain and finally quickly dehydrate and mount in xylene. Apoptotic cells were counted at 100× magnification, by fluorescence microscopy using a Leica DM2000 (Leica Mycrosystems Srl, Solms, Germany) by three independent operators. At least three different fields were analyzed in each sample. The results were expressed as the percentage of apoptotic cells relative to the total number (apoptotic plus unaffected) of cells in the control (100%).

#### 2.11.2. In Vitro

During apoptosis, Annexin-V is exposed from the inner to the outer portion of the membrane, becoming available to bind the Annexin-V/FITC conjugate. Briefly, 2 × 10^4^ cells/cm^2^ were seeded in a 6-well plate and treated as described before. After 24 h cells were detached by gentle tripsinization, then inactivated by medium with 10% FBS and centrifuged for 5 min at 200× *g*. The pellet obtained was suspended in the binding buffer (10 mM Hepes/NaOH, pH 7.40, 140 mM NaCl, 5 mM CaCl_2_), adjusting cell density to 5 × 10^6^ cell/mL. Finally, 1 × 10^6^ cell were incubated with 5 µL Annexin V-FITC and 10 µL PI for 10 min at room temperature. Subsequently, cells were analyzed by flow cytometer using 488 nm excitation and 515 nm band-pass filter for fluorescent detection (on FL1 and FL2 channels for Annexin V-FITC and PI positive cells, respectively). Data were collected for 10,000 cells and analyzed using Flowing Software 2.5.1 (Cell Imaging Core, Turku Centre for Biotechnology. University of Turku and Åbo Akademi University). The reported results correspond to the mean ± SD of the % of cells in apoptosis (early + late apoptosis).

### 2.12. Statistical Analysis

Unless otherwise indicated, all data in vivo are reported as mean ± SD different animals of each experimental group vs. sex and age-matched HFHC and control group. Statistical comparison between diet groups was performed using one way ANOVA and post-hoc Tukey–Kramer’s test. A *p*-value of <0.05 was considered statistically significant.

Data in vitro are expressed as mean ± standard deviation of three independent experiments (biological replicates). Differences between groups were compared by using unpaired Student’s *t* test when variables were normally distributed; conversely, when they were not, the non-parametric Welch’s unpaired test was performed. The level of significance was set at a *p*-value of 0.05. The effect of FFA+SIL was compared vs. FFA-treated cells.

## 3. Results

### 3.1. Effect on the Body, Liver, and Adipose Tissue Weight

The addition of silymarin to the HFHC diet did not induce any change in the body weight and BMI; however, the weight of the perigonadal (epididymal/ovarian) fat pads (visceral fat) was significantly reduced vs. the HFHC group (Table 2). Moreover, in females a reduction in the liver weight was also observed. After the switch from HFHC to HFHC + SIL diet, a reduction in the food intake of 35%gr (equivalent to 14% Kcal) in males and of 28%gr (7% Kcal) in females was observed. As this difference is marginal, we exclude a differential food intake as possible effector of the observed beneficial effects. Interestingly, the switch of diet from HFHC to CTRL completely restored the normal values of all the parameters in both genders (except for males’ body weight, which was only slightly reduced), whereas CTRL diet supplementation with silymarin (HFHC→CTRL + SIL) did not show additional effects to those observed due to the change of diet.

### 3.2. Effect on Glucose Homeostasis

Significant alterations of the glycemia, insulinemia, and thus the HOMA-IR, were observed only in the HFHC males; females show only hyperglycemia with no insulin resistance (IR). The addition of silymarin to the HFHC diet significantly reduced the glucose levels. In spite of the hypoglycemic properties of the HFHC + SIL, reversion of IR was not achieved mainly because the level of insulin were unchanged. The switch of the diet from HFHC to CTRL improved all the parameters involved in the glucose homeostasis, by restoring the basal levels (Table 3) independently from the addition of silymarin.

### 3.3. Effects on Lipid Profile

**Figure 1:**
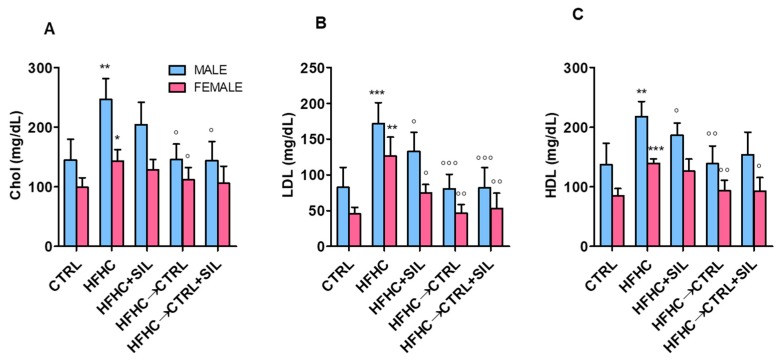
Effect of silymarin-containing food on the lipid profile. The amount of (**A**) total cholesterol; (**B**) LDL cholesterol; and (**C**) HDL cholesterol was measured in all the experimental groups. * *p* < 0.05 vs. CTRL; ** *p* < 0.01 vs. CTRL; *** *p* < 0.001 vs. CTRL and ^°^ *p* < 0.05 vs. HFHC; ^°°^ *p* < 0.01 vs. HFHC; ^°°°^ *p* < 0.001 vs. HFHC.

As previously described [29], HFHC diet induces alterations in the blood lipid profile both in males and females by increasing total cholerterol, LDL, and HDL. The addition of silymarin to the HFHC diet had no effects in the total cholesterol concentration. However, it significantly reduced the LDL levels in both genders, and HDL only in males. (Figure 1A–C). The diet change (HFHC→CTRL), restored the normal lipid profile observed in the control group. Silimarin-enriched CTRL diet did not induce additional benefits.

### 3.4. Effect on Transaminases Activity

**Figure 2:**
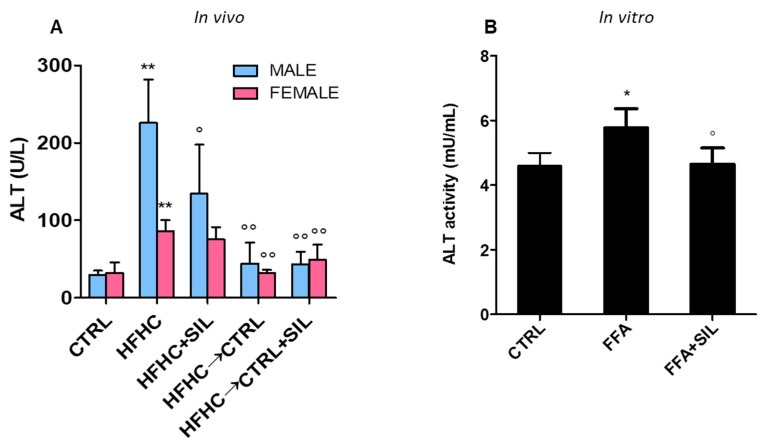
Assessment of the ALT activity. The ALT activity was measured (**A**) in vivo in all the experimental groups and confirmed (**B**) in vitro on fat-laden human hepatocytes (HuH7). * *p* < 0.05 vs. CTRL; ** *p* < 0.01 vs. CTRL and ^°^ *p* < 0.05 vs. HFHC; ^°°^ *p* < 0.01 vs. HFHC.

Aspartate aminotransferase (AST) and alanine aminotransferase (ALT) are surrogate markers used to assess liver damage. In our model, HFHC diet induced a peak in the activity of ALT both in males and females (Figure 2A), whereas AST activity was unchanged (data not shown). The addition of silymarin to HFHC diet showed a statistically relevant reduction in ALT levels only in males. The diet switched to CTRL completely restored the basal activity levels, and the silymarin supplementation did not show a synergic effect. To confirm the effect of silybin on the hepatocyte, AST, and ALT were measured in the culture medium upon exposure to both to FFA and FFA+SIL. Similarly, increase of ALT was induced by FFA and the co-treatment with silybin exerted a protective effect vs. FFA by reducing the amount of ALT (1.24 ± 0.5-folds, *p* < 0.05 vs. FFA) (Figure 2B). In line with the in vivo observation, AST levels were also unchanged in vitro (data not shown).

### 3.5. Hepatic Histological and Molecular Features Upon Silymarin Supplementation

**Figure 3:**
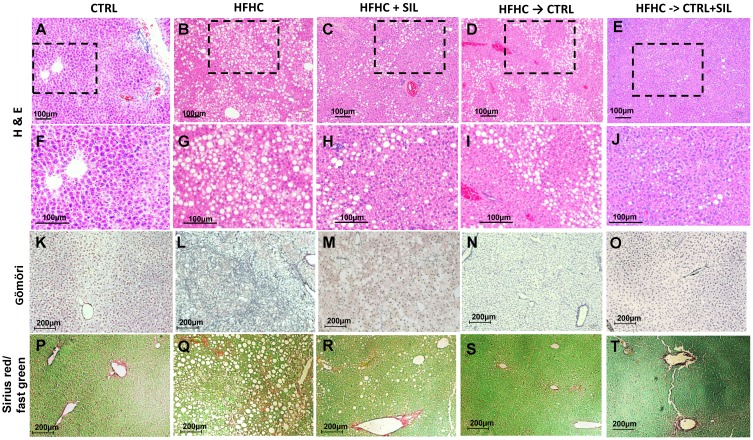

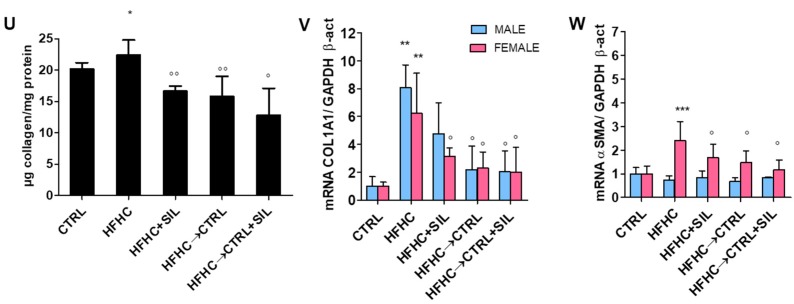
Hepatic histological and molecular features upon silymarin supplementation. Representative slices of liver samples stained with hematoxylin-eosin staining (**A**–**E**), with higher magnification of the dashed area (**F**–**J**). Gӧmӧri Staining for fibrosis assessment (**K**–**O**). Collagen staining was done by Sirius Red/Fast Green staining (**P**–**T**). The µg of collagen was quantified in all groups and normalized by mg/protein (**U**). The mRNA gene expression of *Col1a1* (**V**) and *α-Sma* (**W**) were was assessed by RT-PCR. * *p* < 0.05 vs. CTRL; ** *p* < 0.01 vs. CTRL; *** *p* < 0.001 vs. CTRL and ^°^ *p* < 0.05 vs. HFHC; ^°°^ *p* < 0.01 vs. HFHC; Hepatic histological and molecular features upon silymarin supplementation.

In our model we observed that HFHC diet induced steatosis both in males and females, with a predominant panacinar location in males and azonal in females (Table 4, Figure 3A–J). All males also presented microvescicular steatosis. The addition of silymarin to the HFHC diet had a modest effect in the reduction of the steatosis score in males, and no effect in females. This observation was in agreement with the in vitro experiments where FFA + SIL did not shown differences in the intracellular content vs. FFA treated cells (data not shown). The histological scoring of the portal/lobular inflammation showed no changes in males, whereas in females silymarin seems to reduce the lobular inflammation. As for the other parameters, diet switch from HFHC to CTRL (HFHC→CTRL) (independently from the supplementation with silymarin) restored the basal levels observed in the control group.

The most interesting finding of our study is the effect of the addition of silymarin to the HFHC diet in the regression of fibrosis. While a mild periportal fibrosis (score 1a) was also observed in controls, the HFHC diet group showed a worsening of the fibrosis stage towards a portal/periportal fibrosis (score 1c) in 40% of males and 60% of females. The group of HFHC + SIL in both genders showed a reduction of fibrosis with a predominant score of 1a in the majority of the animals (Table 4. Figure 3K–O). Subsequently, the collagen content in the liver slices was quantified by Sirius Red/Fast Green staining (Figure 3P–T) As shown in Figure 3U we observed a 1.3-folds reduction in the collagen content in the HFHC + SIL group vs. HFHC diet in both genders. The histopathological findings were confirmed further by the molecular analysis of the hepatic collagen (*Col1a1*) gene expression. Both males and females showed an increased expression of collagen induced by HFHC diet, whereas in both genders a two-fold reduction was observed by the addition of silymarin (Figure 3V). Interestingly, we observed that even if collagen was upregulated in both genders exposed to HFHC diet, only females showed a sustained a*-Sma* up-regulation (hepatic stellate cells (HSC) activation marker). Its expression in this group was restored to the basal levels by silymarin enrichment (Figure 3W). This finding suggests that the pro-fibrotic environment is still active in females, whereas in males is likely that all HSC cells have become myofibroblasts.

**Figure 4:**
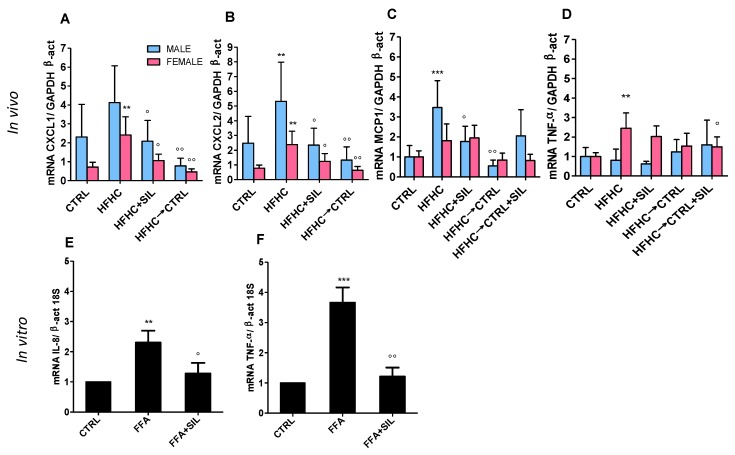
Molecular analysis of hepatic pro-inflammatory genes. The expression of pro-inflammatory mediators was assessed both in vivo (**A**–**D**) and in vitro (**E**–**F**). ** *p* < 0.01 vs. CTRL; *** *p* < 0.001 vs. CTRL and ^°^ *p* < 0.05 vs. HFHC; ^°°^ *p* < 0.01 vs. HFHC.

Molecular analysis of pro-inflammatory mediators showed that HFHC + SIL is effective to decrease the gene expression both of *Cxcl-1* and *Cxcl-2* (murine homologous of human *IL8*) (Figure 4A,B); *Mcp-1* (only in males) (Figure 4C) while has no effect on the expression of *Tnf-α* either in males or in females (Figure 4D). The in vitro data showed that hepatocytes co-treated with FFA+SIL showed *IL-8* and *TNF-α* expression returned to basal levels (Figure 4E–F). Interestingly, hepatocyte expression of *MCP-1* was under the detection limit and was not induced by FFA exposure (data not shown). As mentioned before, also in this case the switch of HFHC to CTRL diet (independently of the presence of silymarin) restored the basal levels of all the parameters under study.

### 3.6. Antioxidant Properties

**Figure 5:**
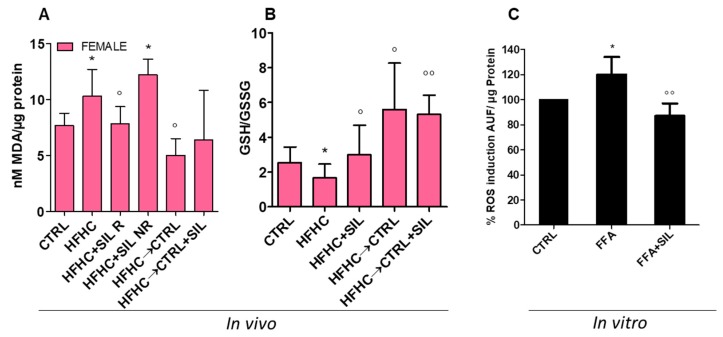
Assessment of antioxidant properties of silymarin formulation. The redox state was calculated in vivo as the amount of lipoperoxides (MDA) (**A**) and the cellular antioxidant properties measured as the glutathione ratio (GSH/GSSG) (**B**). Whereas, in vitro were measured the total amount of reactive oxygen species (**C**). * *p* < 0.05 vs. CTRL; ^°^ *p* < 0.05 vs. HFHC; ^°°^ *p* < 0.01 vs. HFHC.

The production of reactive oxygen species (ROS) is tightly associated with the hepatic manifestation of the metabolic syndrome, and is considered one of the major triggers of the progression of the damage. ROS generation was assessed in liver homogenates through the quantification of MDA and GSH/GSSG ratio. A significant MDA increment was observed only in HFHC females (Figure 5A), while males did not show any difference (data not shown). This alteration was mirrored also by the GSH/GSSG ratio that followed the same pattern (Figure 5B). The addition of silymarin to the HFHC diet induced a partial reduction of MDA levels vs. the HFHC group, even if the reduction was not statistically significant (*p* = 0.06). Interestingly, within this group (HFHC + SIL females) it was possible to identify two sub-groups: responders (HFHC + SIL R) and non-responders (HFHC + SIL NR) (Figure 5A). Addition of silymarin to the HFHC diet restored the hepatic redox equilibrium as indicated by GSH/GSSG ratio. The ability of silybin to counteract the FFA-induced oxidative stress was observed also in vitro. After 1 h silybin promotes a reduction (27.5 ± 10.0%, *p* < 0.05) in ROS generation (Figure 5C) whereas no differences were observed at 24 h, confirming oxidative stress in an early cellular response.

Also in this case, switch from HFHC to control diet (CTRL) led to a reduction of MDA levels, decreased the lipid peroxidation, and improved the GSH/GSSG ratio, confirming the antioxidant benefits of this therapeutic approach. Also, in this case, no synergic effect was observed with the enrichment of silymarin in the HFHC→CTRL + SIL group.

### 3.7. Anti-Apoptotic Effect

**Figure 6:**
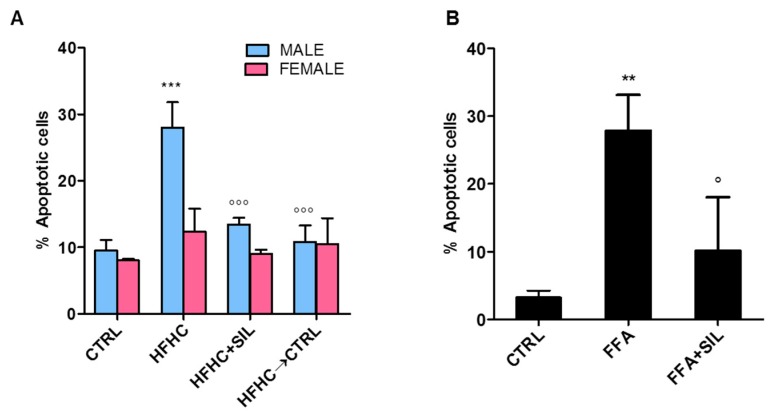
Effect of silymarin formulation on apoptosis. The number of cells undergoing apoptosis was counted in vivo (**A**) and in vitro (**B**). ** *p* < 0.01 vs. CTRL; *** *p* < 0.001 vs. CTRL; ^°^ *p* < 0.05 vs. HFHC; ^°°°^ *p* < 0.001 vs. HFHC.

Apoptosis is one of the events that participates in the progression of simple steatosis to steatohepatitis [44]. In our model we observed an induction of apoptosis (28% ± 4%, *p* < 0.001) in the HFHC vs. CTRL group (Figure 6A) in males whereas females did not present changes in the number of apoptotic cells (data not shown). The addition of silymarin significantly reduced the cells in apoptosis restoring the CTRL group levels. Also in this case the switch from HFHC to CTRL diet fully reverted the damage. In line with this observation, cells exposed to FFA showed a significant increment in apoptosis (27.85 ± 5.29%, *p* < 0.01 vs. CTRL cells). Co-treatment of FFA+SIL reduced the number of apoptotic cells (10.12 ± 7.9%, *p* < 0.05 vs. FFA), restoring the basal levels as CTRL cells (Figure 6B). These data indicate that silybin effectively counteracts the lipoapoptotic mechanism.

## 4. Discussion

Over the last 40 years, the obesity outbreak has been challenging the worldwide care system [45]. It has been reported that obesity is tightly associated with the incidence of NAFLD [46,47]. NALFD is the most common cause of liver disease in the pediatric population with a prevalence of 10.2% in Asians; 1.5% in Blacks; 11.8% Hispanic; and 8.6% in Caucasians [48]. While in adults NAFLD is considered a benign, potentially reversible, and slow progressive liver disease, in children it can progress rapidly to NASH reaching end-stage liver disease during adolescence [48,49]. In spite of this alarming evidence, to date there are no approved pharmacological therapies for NAFLD neither in children nor in adults. The best and most effective option still relies on lifestyle modifications including nutrition [7], exercise [6], and dietary supplements [50]. However, patients’ compliance is very low towards these approaches and usually there is a poor long-term success rate [51]. The use of dietary supplements as complementary and alternative medicine represents an appealing and fertile field of study; unfortunately, data published so far did not fulfill the expectations (extensively reviewed elsewhere [28]).

Silymarin extract has been used since ancient times in the treatment of various liver disorders [11,12]. Based on this evidence, silymarin has become an interesting therapeutic candidate for NAFLD. Data from animal models are promising, and it has been demonstrated that silymarin exerted positive effects in improving many of the pathological features of NAFLD. Unfortunately, the translation to the human scenario is unfeasible mainly because the studies reporting beneficial effects were conducted in animals where silymarin had been administrated intraperitoneal or by gastric gavage (reviewed elsewhere [27]). The oral administration of silymarin is hampered by low solubility in water, poor intestinal absorption and, consequently, its low and poorly predictable bioavailability [52]. The combination of milk thistle extracts with solubilizing substances can improve this aspect. In the present study, we explored the effects of the addition of silymarin to the western diet (HFHC), thus administrated orally. For this purpose, a special food formulation was prepared taking advantage of the ingredients already present into the diet to facilitate silymarin solubility.

One of our main goal was to assess (as physiologically as possible) the effect of the silymarin-enriched food in an obesogenic condition, using a juvenile murine model of NASH. Conversely to other studies in which adult mice were used, in the present study the animals were fed immediately after weaning for a total of 20 weeks (equivalent to human 3–30 years old [53]). Since we previously reported [29] a sex-related difference in NAFLD onset and in progression to NASH, in this work we investigated a possible sex dimorphism in the therapeutic response.

The HFHC + SIL diet improved many of the crucial parameters involved in the progression of NAFLD. For instance, silymarin-enriched food reduced the visceral fat in both genders, with no effect on the total body weight. In agreement with our observation, it has been has been recently reported [54] that dietary silymarin supplementation significantly reduced the elevated intraperitoneal fat index in grass carp fed with high-lipid diets. This study also showed that silymarin decreases the expression of the adipogenic genes *Ppar* γ, *C/Ebp* α, *Srebp1c*, *Fas*, *Scd1*, and *Lpl*. HFHC + SIL also improved the hepatomegaly in females, whereas in males reduced the ALT activity. Our study demonstrates that HFHC diet induced IR only in males. Silymarin slightly reduced the glucose but not insulin level, and thus not improving IR. This observation leads to questioning the real efficacy of silymarin in the reversion of the alterations in glucose homeostasis.

The most evident effect of HFHC + SIL on lipid profile was the reduction of LDL-cholesterol in both genders, with no statistically relevant changes in the total cholesterol levels. This observation is particularly interesting due to the involvement of LDL cholesterol in arteriosclerosis and cardiovascular disease [55]. There is a popular designation of HDL as ‘the good cholesterol’ due to the inverse correlation with coronary heart disease risk. However, paradoxically, diets high in saturated fat and cholesterol which increase atherosclerosis risk also raise HDL levels [56]. This increase in HDL was also observed in the HFHC group, whose levels were restored to control values after life-style changes (group switched from HFHC to CTRL). The addition of silymarin to the HFHC diet (HFHC + SIL) induced a reduction of HDL vs. the values observed in the HFHC littermates.

Even if in the present study we did not perform bioavailability tests, we did observe hepatoprotective effects by the oral consumption of the formulation of western diet containing silymarin, suggesting that silymarin was absorbed. These observations were further confirmed in vitro by the direct exposure of human hepatocytes to silybin+FFA.

We observed that HFHC + SIL diet exerted an anti-inflammatory effect inducing a reduction in the hepatic expression of some cytokines (*Cxcl-1* and *Cxcl-2* (murine *IL-8* homologous) in both genders, and *Mcp-1* only in males. These changes had no relevant effects on the histological inflammatory activity score. The anti-inflammatory effect of silybin was confirmed in vitro with a similar pattern of downregulation in the gene expression of *IL-8* and *TNF-α* on the hepatocytes. Since levels of *MCP-1* in hepatocytes were under the detection limit, it is tempting to speculate that the downregulation of this cytokine observed in the males might be a consequence of the effect of the silymarin on other hepatic cell types. It is well-known that *MCP-1* expression is related to the number of monocytes infiltrated and is an important mediator for the progression to NASH [57]. Also IL-8, once secreted, acts as a potent chemoattractant for neutrophils and leukocytes recruitment [58].

Oxidative stress is recognized as a driving force in the onset and the progression of NAFLD.

Since the HFHC diet induced hepatic oxidative stress only in females [29], the eventual anti-oxidant properties of the silymarin-enriched formulation were assessed only in females. Some females reverted the amount of hepatic lipoperoxides (MDA) and the entire group showed a GSH/GSSG ratio significantly improved by the addition of silymarin to the HFHC diet. Silybum marianum extract has high polyphenolic content that leads to a well-recognized antioxidant effect through free radical scavenging [59]. The reduction of ROS generation determines the attenuation of lipid peroxidation, observed in our model with a partial MDA decrease and with the increase of hepatic GSH content. It has been demonstrated that silymarin is able to regulate GSH content with organ-specific action [60]. Based on the short half-life of silymarin, the liver is its main target, the principal site of silymarin accumulation and the starting point for a cyclic transportation via entero-hepatic circulation. As a result, the liver (together with the stomach and the intestine) is the tissue receiving the higher silymarin amount that justifies the large increase in GSH concentration. In line with these findings, in vitro data clearly showed how the co-treatment of silybin+FFA is able to counteract the ROS generation induced by FFA. All together these data indicate that silymarin formulation is able to reduce the HFHC-induced oxidative stress.

We observed a clear gender difference in the induction of lipo-apoptosis. HFHC diet had a potent pro-apoptotic effect in males whereas in females this feature was absent. Hence, the assessment of the anti-apoptotic effect of the silymarin formulation was only possible in males, in which HFHC + SIL diet significantly reduced the number of apoptotic cells. It has been extensively reported the activity of silymarin on the activation of pro-caspase 3 [61], involved in the binding to death receptor of Fas/tumor necrosis factor receptor family and formation of death inducing signal complex [62]. The anti-apoptotic effect of silymarin was also confirmed in vitro. Human fat-laden hepatocytes showed an increased generation of ROS that was successfully reduced by silymarin treatment.

We reported previously [29] that our juvenile model of NASH presented a time-dependent progressive fibrosis with a histological score of 1c–2 after 16 weeks of HFHC diet. To our knowledge, this is the only DIO model showing this degree of liver fibrosis in such a short time frame. The most relevant finding of the present study is the effect of HFHC + SIL on this feature. Herein we observed an improvement in the histological liver fibrosis score in both genders, it is likely that with longer exposure times to silymarin formulation the extent of improvement would be higher. This observation was also confirmed in terms of collagen gene expression and extracellular protein deposition. The onset of a sustained inflammation and oxidative stress typical of NASH increases the risk of progression to advanced fibrosis. In the cytosol, the release of cytokines initiates intracellular signaling (via NF-kB) with the final activation of HSC [63]. Silymarin is able to counteract the inflammatory milieu mainly by suppressing NF-kB activation [64] and thus prevent the following pro-fibrogenic processes. This finding is particularly relevant as fibrogenesis is a crucial step in the progression from NAFL to NASH and, consequently, in the severity of the liver disorder. Furthermore, it has been recently described that no other histological features but fibrosis are associated with the long-term outcomes of patients with NAFLD [65]. For these reasons, the silymarin food formulation used in this study presents many promising perspectives.

In the present study, we assessed also the effect of lifestyle changes in the reversion of the pathological features induced by HFHC diet. In line with previous data, the diet switched from HFHC to CTRL diet improved/reverted all the parameters under study. We also explored if silymarin supplementation would provide a synergic beneficial to lifestyle changes, however our data showed that the overall improvement was mainly due to diet changes independent from the silymarin supplementation.

## 5. Conclusions

The novelty of the present work relies on the addition of silymarin to the western diet as an alternative approach to counteract/revert the damage induced by the excess in the consumption of fat and sugars. The experimental model used represents a diet-induced model of obesity able to reproduce many of the features involved in the onset of NAFLD. The particularity of this experimental model is that it covers the ages from the weaning to the adulthood. Translated to humans, it covers the range from 3 to 30 years old, which represents the period where the incidence of obesity is reaching epidemic values [66]. Although the beneficial effect of the formulation present a sexual dimorphism, silymarin was able to improve the liver condition (especially fibrosis) both in males and females.

The use of silymarin in children has been previously tested in pediatric population in the context of other pathologies, with no reported collateral effects [67,68]. Consequently, silymarin can be safely tasted in this population. Nevertheless, to our knowledge, currently there is no planned clinical trial for pediatric NASH [69].

All together, we consider that the data provided represent a valid strategy for the future development of dietary supplements containing silymarin. These approaches represent an alternative to overcome patients’ low compliance to diet changes in the field of NAFLD/NASH.

## Figures and Tables

**Table 1:** Primer pair sequences.

**Mouse Genes**	**Accession Number**	**Forward**	**Reverse**
*Gapdh*	NM_008084	CCAGTATGACTCCACTCACG	CTCGCTCCTGGAAGATGGTG
*β-Actin*	NM_007393	CCTTCTTGGGTATGGAATCCTGTG	CAGCACTGTGTTGGCATAGAGG
*Dgat-2*	NM_026384	CCAAGAAAGGTGGCAGGA	ATGGGAAAGTAGTCTCGGAAG
*Mcp-1*	NM_011333	TGTGACTCGGACTGTGAT	CATTGAAAGTGTTGAATCTGGAT
*Tnf-α*	NM_013693	AGACCCTCACACTCAGAT	CTTGGTGGTTTGCTACGA
*Cxcl-1*	NM_008176	CATAGCCACACTCAAGAA	GGACACCTTTTAGCATCT
*Cxcl-2*	NM_009140	ACTTCAAGAACATCCAGAG	TTGAGAGTGGCTATGACT
*α-Sma*	NM_007392.2	GGCATCAATCACTTCAAC	TCTGGTCACCTGTATGTA
*Col-1a1*	NM_007742.3	AAGAAGACATCCCTGAAG	ATACAGATCAAGCATACCT
**Human Genes**	**Accession Number**	**Forward**	**Reverse**
*β-Actin*	NM_001101.3	CGCCGCCAGCTCACCATG	CACGATGGAGGGGAAGACGG
*18S*	NR_003286.2	TAACCCGTTGAACCCATT	CCATCCAATCGGTAGTAGCG
*IL-8*	NM_000584	GACATACTCCAAACCTTTCCAC	CTTCTCCACAACCCTCTGC
*TNF-α*	NM_000594	GTGAGGAGGACGAACATC	GAGCCAGAAGAGGTTGAG
*MCP-1*	NM_002982	CCAGTCACCTGCTGTTAT	CAATGGTCTTGAAGATCACA

**Table 2:** Macroscopic features in males and females.

Parameters	MALES					FEMALES				
	Control	HFHC	HFHC + SIL	HFHC→CTRL	HFHC→CTRL + SIL	Control	HFHC	HFHC + SIL	HFHC→CTRL	HFHC→CTRL + SIL
Body weight (g)	29.2 ± 2.6	43.4 ± 1.8 ***	43.7 ± 4.6	34.4 ± 2.6 ^###^	30.3 ± 3.3 ^###^	23.4 ± 1.0	35.4 ± 4.3 **	31.8 ± 3.4	23.5 ± 1.5 ^##^	23.9 ± 1.9 ^##^
BMI (kg/m^2^)	3.2 ± 0.3	4.8 ± 0.2 ***	4.8 ± 0.5	3.7 ± 0.3 ^###^	3.3 ± 0.4	2.9 ± 0.1	4.4 ± 0.5 ***	3.9 ± 0.4	2.9 ± 0.2 ^###^	2.9 ± 0.1 ^##^
Liver weight (g)	0.96 ± 0.08	1.8 ± 0.2 ***	1.8 ± 0.1	1.1 ± 0.1 ^##^	1.00 ± 0.04 ^##^	0.88 ± 0.05	1.2 ± 0.1 *	1.09 ± 0.15 ^#^	0.9 ± 0.1	0.94 ± 0.01 ^##^
Perigonadal Fat Pads (g)	0.98 ± 0.08	2.4 ± 0.3 ***	2.3 ± 0.2 ^#^	1.3 ± 0.28 ^##^	1.0 ± 0.4 ^##^	0.32 ± 0.01	1.5 ± 0.6 **	0.9 ± 0.3 ^#^	0.32 ± 0.09 ^###^	0.27 ± 0.07 ^###^

At the end of the trial the total body, the liver and the perigonadal fat pads weight were measured and the body mass index (BMI) was calculated. * *p* < 0.05 vs. CTRL; ** *p* < 0.01 vs. CTRL; *** *p* < 0.001 vs. CTRL and ^#^ *p* < 0.05 vs. HFHC; ^##^ *p* < 0.01 vs. HFHC; ^###^ *p* < 0.001 vs. HFHC.

**Table 3:** Changes in glucose homeostasis.

Parameters	MALES					FEMALES				
	Control	HFHC	HFHC + SIL	HFHC→CTRL	HFHC→CTRL + SIL	Control	HFHC	HFHC + SIL	HFHC→CTRL	HFHC→CTRL + SIL
Glucose (mg/dL)	164 ± 20	260 ± 24 ***	226 ± 17 ^#^	167 ± 13 ^##^	187 ± 17 ^##^	141 ± 10	193 ± 25 **	181 ± 31	134 ± 6 ^###^	142 ± 12 ^###^
Insulinemia (µU/mL)	1.9 ± 0.4	4.1 ± 1.1 **	3.6 ± 1.3	2.0 ± 1.2 ^##^	1.5 ± 0.7 ^###^	0.9 ± 0.1	2.5 ± 1.7	2.1 ± 0.5	1.3 ± 0.3 ^#^	1.2 ± 0.4 ^#^
HOMA-IR	1.4 ± 0.4	2.8 ± 0.7 *	2.0 ± 0.8	0.6 ± 0.1 ^##^	0.7 ± 0.4 ^##^	0.3 ± 0.004	1.3 ± 1.0	0.9 ± 0.2	0.4 ± 0.1	0.4 ± 0.2

In the table are reported the values of glycemia, insulinemia as well as de HOMA-IR after the trial. * *p* < 0.05 vs. CTRL; ** *p* < 0.01 vs. CTRL; *** *p* < 0.001 vs. CTRL and ^#^ *p* < 0.05 vs. HFHC; ^##^ *p* < 0.01 vs. HFHC; ^###^ *p* < 0.01 vs. HFHC.

**Table 4:** Histological features.

	MALES					FEMALES				
Parameters	CTRL	HFHC	HFHC + SIL	HFHC→CTRL	HFHC→CTRL +SIL	CTRL	HFHC	HFHC + SIL	HFHC→CTRL	HFHC→CTRL +SIL
Steatosis Grade (0–3)	2 (100%)	3 (100%)	3 (60%) 2 (40%)	2 (50%) 1 (50%)	1 (60%) 2 (40%)	1 (33%) 2 (66%)	1 (20%) 2 (80%)	2 (100%)	1 (75%) 2 (25%)	2(100%)
Location (score 0–3)	3 (100%)	3 (40%) 0 (60%)	3 (60%) 2 (40%)	3 (75%) 2(25%)	2 (100%)	3 (100%)	0 (20%) 2 (60%)	0 (33.3%) 2 (66.6%)	2 (100%)	2 (100%)
Portal Inflammation (score 0–1)	1 (100%)	0 (100%)	0 (88%) 1 (12%)	1 (50%) 0 (50%)	0 (100%)	0 (100%)	0 (60%) 1 (40%)	0 (50%) 1 (50%)	0 (25%) 1 (75%)	0 (100%)
Lobular Inflammation Score (0–3)	0 (20%) 1 (80%)	0 (80%) 1 (20%)	0 (40%) 1 (60%)	1 (75%) 0 (25%)	1 (100%)	1 (66%) 0 (33%)	0 (40%) 1 (40%) 2 (20%)	1 (100%)	0 (25%) 1 (75%)	0 (100%)
Fibrosis Stage (0–4)	1a (80%)	1a (60%) 1c (40%)	1a (50%) 1c (25%) 0 (25%)	1a (50%) 1c (25%) 0 (25%)	1a (100%)	1a (83.3%) 1b (16.6%)	1a (40%) 1c (60%)	1a (83.3%) 1b (16.6%)	0 (25%) 1a (25%) 1b (25%) 1c (25%)	1a (100%)

Histological analysis according to Brunt’s classification. Data is expressed as score/grade and between brackets the occurrence.
